# Hashimoto Encephalopathy With Status Epilepticus

**DOI:** 10.7759/cureus.11857

**Published:** 2020-12-02

**Authors:** Aleksandra Sliwinska, Perry Fumuso, Bryan Stringer, Muhammad Ansar, Jennifer Baldwin

**Affiliations:** 1 Department of Medicine, University of Connecticut School of Medicine, Farmington, USA; 2 Department of Endocrinology and Metabolism, University of Connecticut School of Medicine, Farmington, USA

**Keywords:** hashimoto encephalopathy, status epilepticus, seizure, hypothyroidism, hashimoto’s

## Abstract

Hashimoto encephalopathy (HE) is a heterogenous neurological syndrome that can manifest with encephalopathy, seizures, headaches, and variable neuropsychiatric disturbances. The underlying mechanism remains unclear; however, autoimmune pathogenesis is suspected due to its association with autoimmune thyroid disease, high titers of anti-thyroid antibodies, and quick response to steroid therapy. We report a 59-year-old female patient with a remote history of hypothyroidism who presented with status epilepticus and complaints of chronic headaches and cognitive impairment. The presence of sharp frontal waves was identified on her EEG. The patient was initially started on anti-epileptics only; however, her headaches and memory loss escalated, further diagnostic workup was pursued, which revealed high anti-thyroid peroxidase antibodies with normal thyroid function tests. The only cerebrospinal fluid (CSF) abnormality noticed was an elevated protein concentration. MRI showed non-specific right frontal lobe pial enhancement. Remaining infectious, rheumatologic, and neurologic testing was unremarkable. The patient was started on a steroid regimen with successful resolution of symptoms and return of cognitive baseline. Hashimoto’s encephalopathy is a diagnosis of exclusion; however, it should be considered in patients with high titers of anti-thyroid antibodies and neurological symptoms that cannot be explained by thorough infectious, metabolic, and autoimmune testing. It is essential to recognize this neurological entity as fast clinical improvement may be achieved with steroids and other immunotherapies.

## Introduction

Hashimoto encephalopathy (HE), also known as steroid-responsive encephalopathy with autoimmune thyroiditis (SREAT), is a clinically heterogeneous neurological syndrome that is commonly associated with Hashimoto thyroiditis [1]. Due to its possible underrecognition and rarity, the literature on Hashimoto encephalopathy remains sparse. Given non-specific testing modalities and clinical manifestations that overlap with more commonly identified pathologies, establishing a diagnosis can be challenging and requires extensive investigation to exclude other neurological, rheumatological, and infectious conditions. HE has been described as a diagnosis of exclusion. Some experts dispute whether HE is a distinct clinical entity.

By definition, the presentation of Hashimoto encephalopathy consists of nonspecific alterations in mental status and various neurological signs and symptoms; these include but are not restricted to seizure, ataxia, myoclonus, headache, and/or psychiatric disturbances. Behavioral and cognitive changes are the most commonly reported clinical features [2]. Seizures have been described as a common presentation, with 60-66% of patients experiencing it as part of their constellation of signs and symptoms [3]. Encephalopathy may be progressive or fluctuating without a particular pattern [1, 3].

Given the lack of recognized diagnostic criteria, definitively diagnosing Hashimoto encephalopathy can be challenging. Abnormal thyroid antibodies, especially anti-thyroid peroxidase antibodies (anti-TPO), are found in most cases [1]. Due to the low specificity of plasma anti-thyroid antibodies, cerebrospinal fluid (CSF) is often analyzed, and MRI of the brain is performed. Most patients respond well to steroid therapy with complete resolution of symptoms, although patients may have a relapsing-remitting course [1]. Here, we discuss an interesting case of Hashimoto encephalopathy in a patient who presented with constant headache and status epilepticus.

## Case presentation

A 59-year old Caucasian, left-handed woman with a past medical history of hypertension and hyperlipidemia presented to an outside hospital with seizures. She was witnessed at work to have involuntary turning of her head to the left with vocalization (incomprehensible sounds) followed by tonic-clonic seizures. After this episode, she did not regain consciousness prompting further evaluation at the hospital for status epilepticus. Upon arrival, the patient continued to have seizure-like activity; thus, she received a loading dose of 10 mg of midazolam and 1 g of levetiracetam, which resulted in cessation of seizure-like activity and a return to baseline mental status and consciousness. She described a one-month history of mild, dull, constant headache in the bilateral temporal area radiating to the jaw, not associated with nausea, vomiting, or photophobia. The patient did not endorse fevers, chills, or weight loss. She had never carried a formal diagnosis of a seizure disorder and was not using alcohol. Initial lactate was high, which further normalized, and spot EEG did not show epileptiform waves. Brain MRI and head CT were unremarkable, and after a short period of hospitalization, she was discharged home on levetiracetam 500 mg twice daily with instructions to follow-up with neurology. During this brief interval, she experienced unusual behavior, confusion, and short-term memory impairment. She underwent a 24-hour ambulatory EEG showing focal slowing over her right hemisphere with right sharp frontal waves occurring in a quasi-periodic fashion at the frequency of 1Hz. No definite seizure activity was seen. Given this abnormal EEG, with concomitant mental status changes, she was encouraged to return to the emergency department (ED) for further investigation.

Upon arrival, the patient was hemodynamically stable with a blood pressure of 144/89 mmHg, heart rate (HR) 92 bpm, and a temperature of 99.1° F. She complained of mild confusion, short-term memory impairment, and constant mild headache. On general examination, the patient was alert, oriented, and fluent, and able to follow multistep commands. She was able to recall only two out of six words from three short phrases after nine minutes without prompting. There was no dysarthria. Cranial nerves were intact bilaterally. The motor exam revealed normal tone and full-strength throughout with normal reflexes. There were no sensation deficits. Neither ataxia nor dysmetria were appreciated, and rapid alternating movements were normal. The patient’s gait was steady with a negative Romberg test. The remainder of the systemic examination was unremarkable.

**Figure 1:**
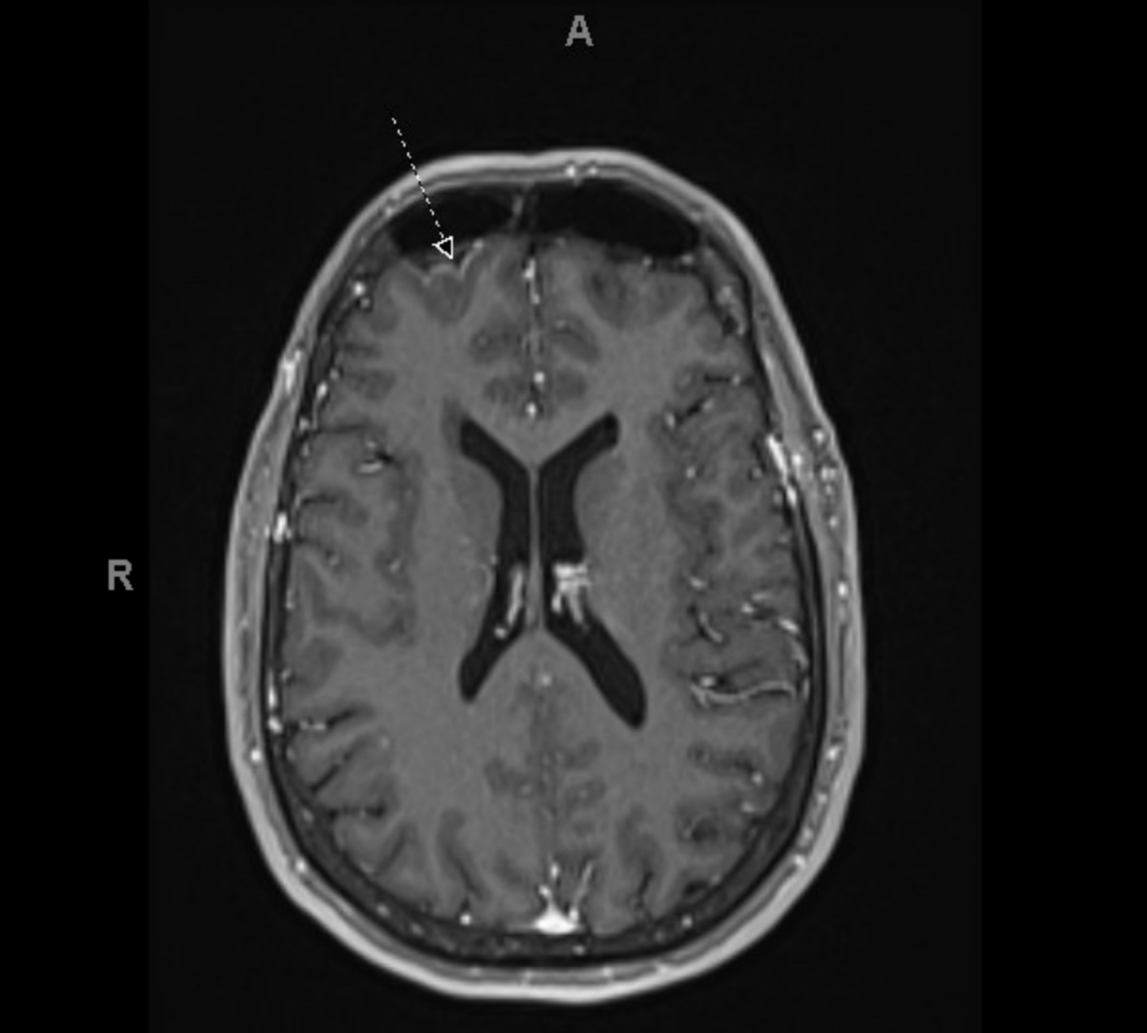
Brain MRI, FLAIR image, axial view: right frontal lobe pial enhancement (arrow) FLAIR - fluid-attenuated inversion recovery

Twenty-four-hour EEG showed focal slowing over the right hemisphere with right more than left sharp waves at times occurring in quasi-periodic fashion at a frequency of 1-2Hz. The presence of frontal sharp waves was identified on her EEG, which are often correlated with increased risk of focal onset seizure. MRI of the brain demonstrated subtle indistinctness of the CSF adjacent to the anterior pole of the right frontal lobe with subtle pial enhancement on post-contrast imaging (Figure 1). Infectious and rheumatologic workup was negative (Table 1). The patient underwent a lumbar puncture (LP), which demonstrated clear CSF with 71 white blood cells (WBC) cells/m3, 3 red blood cells (RBC) cells/m3. Glucose concentration in CSF was normal; however, the protein was elevated to 51 mg/dl (ref: 15-41 mg/dl). The meningitis and encephalitis panel was negative (Table 1). Autoimmune and paraneoplastic testing remained unremarkable. CT of the chest and abdomen was performed to evaluate occult malignancy but did not identify any concern areas. A hyper-vascular, enlarged thyroid gland was incidentally noted on the CT scan, which prompted endocrine evaluation.

**Table 1:** Infectious, autoimmune, and paraneoplastic workup CSF - cerebrospinal fluid; RPR - rapid plasma reagin; VDRL - venereal disease research laboratory; HBV - hepatitis B virus; HCV - hepatitis C virus; ANA - antinuclear antibody; ANCA - antineutrophil cytoplasmic antibody; PCR - polymerase chain reaction; CMV - cytomegalovirus; HSV - herpes simplex virus; HHV - human herpes virus; HPV - human papillomavirus; VZV - varicella zoster virus; AGNA - anti-glial nuclear antibody; ANNA - antineuronal nuclear antibody; CRMP - collapsin response-mediator protein; IgG - immunoglobulin G; PCA - Purkinje cell antibody; AMPA-R-Ab - Alpha-Amino-3-Hydroxyl-5-Methyl-4-Isoxazolepropionic acid receptor Ab; CASPR2 - contactin-associated protein-2; DDPX - dipeptidyl-peptidase–like protein 6; GAD65 - glutamic acid decarboxylase 65; GFAP IFA - glial fibrillary acidic protein immunofluorescence assay; LGI1 - leucine‐rich glioma‐inactivated 1; mGluR1 - metabotropic glutamate receptor 1; NMDA-R - anti-N-methyl D-aspartate receptor; LCM - lymphocytic choriomeningitis.

Serum	CSF
Lyme disease antibodies, RPR, VDRL, HIV, HBV and HCV panel, ANA, ANCA, Anti-Ro, Anti-La, Anti-Sm, dsDNA Ab, C3, C4.	PCR: Escherichia coli, Haemophilus influenzae, Neisseria meningitidis, Listeria monocytogenes, Streptococcus agalactiae, Streptococcus pneumoniae, CMV, enterovirus, HSV-1, HSV-2, HHV-6, HPV, VZV, Cryptococcus neoformans/gattii. Antibodies: Amphiphysin Ab, AGNA-1, AGNA-2, ANNA-1, ANNA-2, ANNA-3, CRMP-5-IgG, PCA-Tr, PCA-1, PCA-2, AMPA-R-Ab, CASPR2-IgG, DDPX Ab, GABA-B-R Ab, GAD65 Ab, GFAP IFA, LGI1-IgG, mGluR1 Ab, NMDA-R Ab, LCM virus.

**Figure 2:**
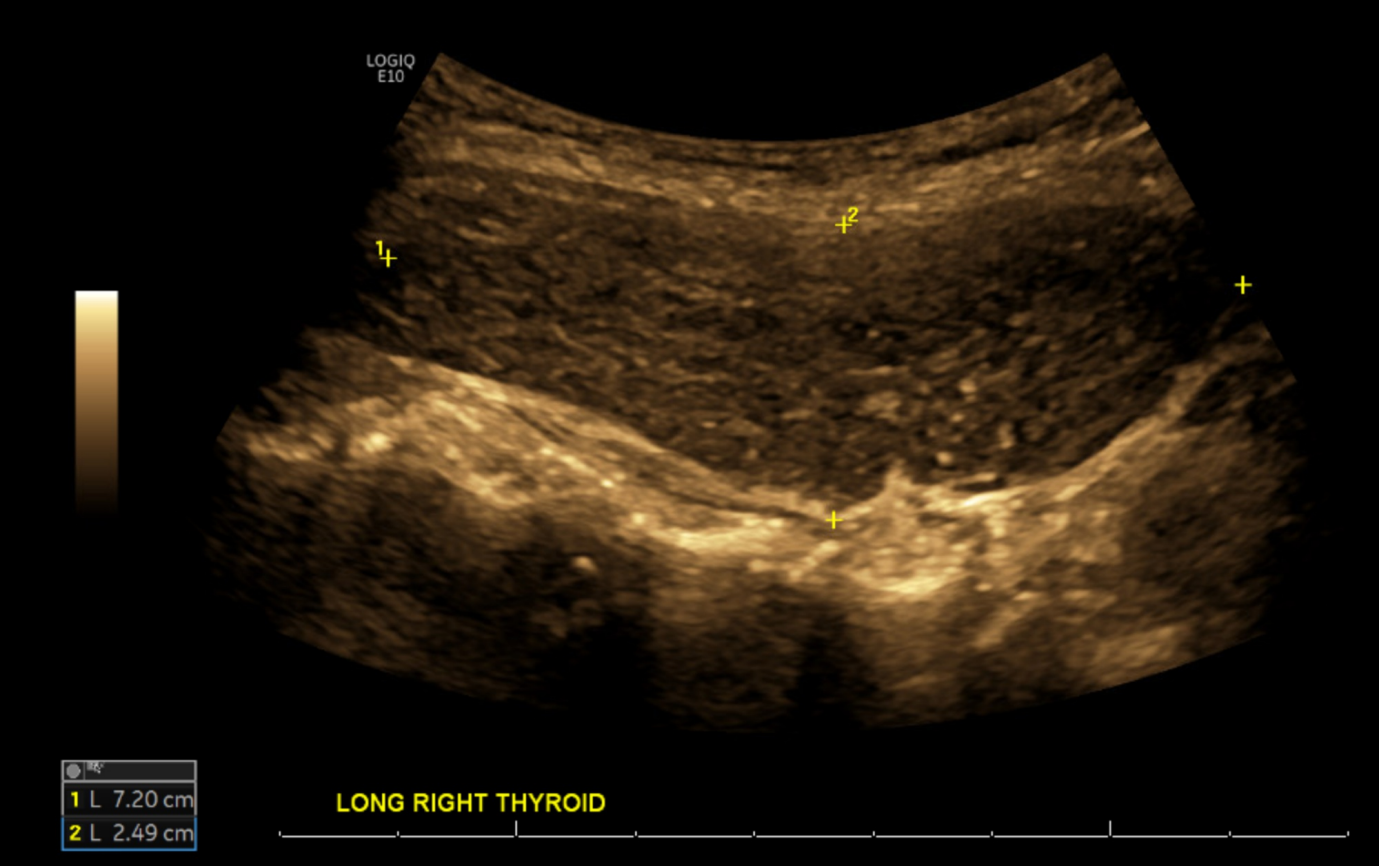
Thyroid ultrasonography, longitudinal view: enlarged, heterogeneous right thyroid lobe measuring 7.2cm x 2.49cm

**Figure 3:**
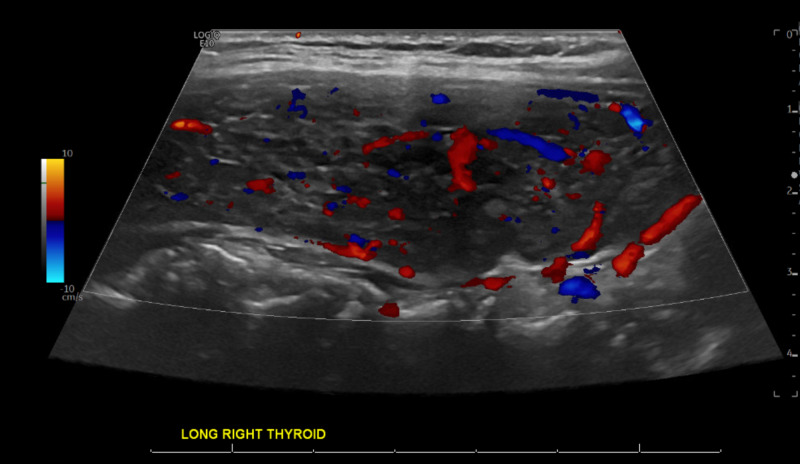
Thyroid doppler ultrasonography, longitudinal view: increased asymmetric vascularity in the right thyroid lobe indicative of Hashimoto thyroiditis

Given the thorough exclusion of more common diagnoses, the investigation turned toward more rare disease entities. Upon further questioning, the patient endorsed a remote history of hypothyroidism and treatment with thyroid replacement therapy; however, she remained off levothyroxine supplementation due to the euthyroid state. As such, Hashimoto's encephalopathy was suspected. The patient was clinically euthyroid without signs or symptoms of hypo- or hyperthyroidism. Thyroid-stimulating hormone (TSH) was 2.57 uU/ml (ref: 0.35-4.94 uU/ml), serum anti-TPO antibodies were elevated to 291.7 U/ml (ref: 0.0 - 60 U/ml). Thyroglobulin (TG) antibody was elevated at 68.2 U/ml (ref: 0-60 U/ml). Thyroid ultrasound showed a heterogeneous thyroid gland with enlargement of the right thyroid and asymmetrically increased vascularity of the right thyroid lobe without focal nodules (Figures 2-3). The patient underwent a repeat LP, which showed CSF anti-TPO Ab at 0.3 IU/ml (ref: 0.0-9.0 IU/ml). Given persistent headaches and mental changes, the patient was started on 1 g methylprednisolone for five days. The patient responded well to the regimen and reported termination of headaches. Given this improvement, she was discharged with an oral steroid taper. A follow-up 24-hour EEG was improved and showed mild focal disturbance of cortical function involving both temporal regions. No seizures, epileptiform discharges, or typical behavioral events were noted. Repeat MRI of the brain did not show any intracranial pathology. The patient was discharged on steroid infusion three times weekly with subsequent oral steroid taper and levetiracetam 1000 mg daily. Upon reassessment in an outpatient setting, the patient showed significant improvement in the cognitive evaluation and denied any further headaches or breakthrough seizures for more than 10 months. 

## Discussion

Hashimoto encephalopathy, also known as steroid-responsive encephalopathy associated with autoimmune thyroiditis, is an uncommon syndrome with a broad spectrum of neurological symptoms. Originally described in 1966, HE has remained a mysterious entity appearing in the literature since, although some experts still question its existence [1, 4]. Given its namesake, Hashimoto encephalopathy has been associated with Hashimoto thyroiditis; however, some pundits argue this relationship may be confounded by elevated titers of anti-thyroid antibodies occurring in up to 20% of healthy individuals [5, 6]. Nonetheless, in a retrospective review, patients diagnosed with Hashimoto encephalopathy had elevated serum anti-TPO antibodies in most of the cases [7]. 

The exact underlying cellular mechanism causing this elusive pathology remains unknown; however, a few hypotheses describing HE pathophysiology have arisen in the medical literature within recent history. One of these hypotheses considers HE an autoimmune disorder due to its association with thyroid antibodies and significant response to steroid therapy. On the other hand, vasculitis and inflammation are thought to play a role in the pathogenesis as perivascular lymphocytic inflammation was found in the brain tissue samples of patients with HE [8]. Despite the elevation of CSF anti-TPO antibodies in most HE cases, they do not seem to play any pathophysiological role in this process. There is no confirmed prognostic value of anti-TPO antibodies within CSF as there is no antigen recognized by these antibodies in the central nervous system [9-11]. This conclusion is given credence, as evidenced by our patient lacking a significant amount of anti-TPO antibodies in her CSF.

HE clinically manifests with acute encephalopathy with different degrees of altered consciousness. As high as two-thirds of patients develop focal or tonic-clonic seizures. Status epilepticus has been reported in 12% of patients. Stroke-like symptoms may occur with focal neurological signs. Psychosis and other behavioral disturbances have also been described [1-3]. The pattern of presentation varies from acutely appearing neurological episodes to progressively diffuse impairment. As in our patient, symptoms slowly progressed from chronic daily headaches to status epilepticus, requiring a hospital stay. The long-term course is also variable and may present as a self-limited syndrome or become progressive and relapsing-remitting [12].

In summary, essential laboratory features include elevated serum anti-TPO antibodies and occasionally anti-TG antibodies. There is no clear correlation between the severity of neurological symptoms and levels of these antibodies neither in serum nor in CSF, however abnormal elevation of anti-TPO antibodies in CSF has been found in 60-72% of patients [7, 9, 13, 14]. Moreover, since serum antibodies may be discovered in up to 20% of healthy individuals, these findings are highly non-specific and non-sensitive. Thyroid function levels vary among patients, with most cases being euthyroid at the time of diagnosis [13]. CSF may be abnormal in up to 85% of patients with HE; in these patients, elevated protein concentration is the most prevalent finding [7]. EEG is often non-specific, with recordings demonstrating generalized background slowing; however, it may be used to monitor response to the treatment [15]. In our patient, EEG has shown significant improvement of focal slowing and the resolution of sharp focal waves following steroid therapy. Imaging is often performed due to concerning neurological symptoms. Retrospectively, up to 49% of patients with HE had non-specific abnormalities on the brain imaging [1].

Hashimoto encephalopathy is a diagnosis of exclusion. Differential diagnoses should be broad and multidisciplinary before the final conclusion. Autoimmune encephalitis, metabolic encephalopathies, demyelinating syndromes, and psychiatric conditions should be considered during investigation [16]. As treatment of HE consists of immunosuppressive therapy, infectious workup should be considered prior to treatment. Since Hashimoto encephalopathy is a rare disorder, no consensus has been established yet regarding steroid dosing and length of treatment. Our patient responded well to high-dose steroids with the resolution of headaches and cognitive improvement. The risk of seizure recurrence is unknown; thus, patients may continue anti-epileptic medications. Several cases reported successful treatment with intravenous immunoglobulins (IVIG) and plasmapheresis, supporting HE's autoimmune etiology [17, 18]. Interestingly, levetiracetam has been shown to have an anti-inflammatory effect and may become an effective alternative treatment for patients who present with seizure episodes or cannot tolerate steroids [19, 20].

## Conclusions

Hashimoto encephalopathy should be suspected in patients with unexplained neurological syndromes and a history of thyroid disorder or elevated anti-TPO antibodies. The incidence of HE may be underestimated due to the non-specificity of symptoms, laboratory testing, imaging, and high prevalence of anti-TPO antibodies in the general populace. Infectious, metabolic, and autoimmune disorders should be excluded before diagnosis. Steroid therapy remains a mainstay of treatment; however, levetiracetam and IVIG have shown promising effects.
